# Supplementary material for: All-at-once RNA folding with 3D motif prediction framed by evolutionary information
Source: Nat Methods. 2025 Oct 3;22(10):2094–106. doi: 10.1038/s41592-025-02833-w (PMC12510878; doi:10.1038/s41592-025-02833-w)
Supplement: Supplementary file 3 — Source code to run CaCoFold-R3D. Data and results. [file 41592_2025_2833_MOESM3_ESM.gz › supplemental_material/Figures/Figure_2.pdf]

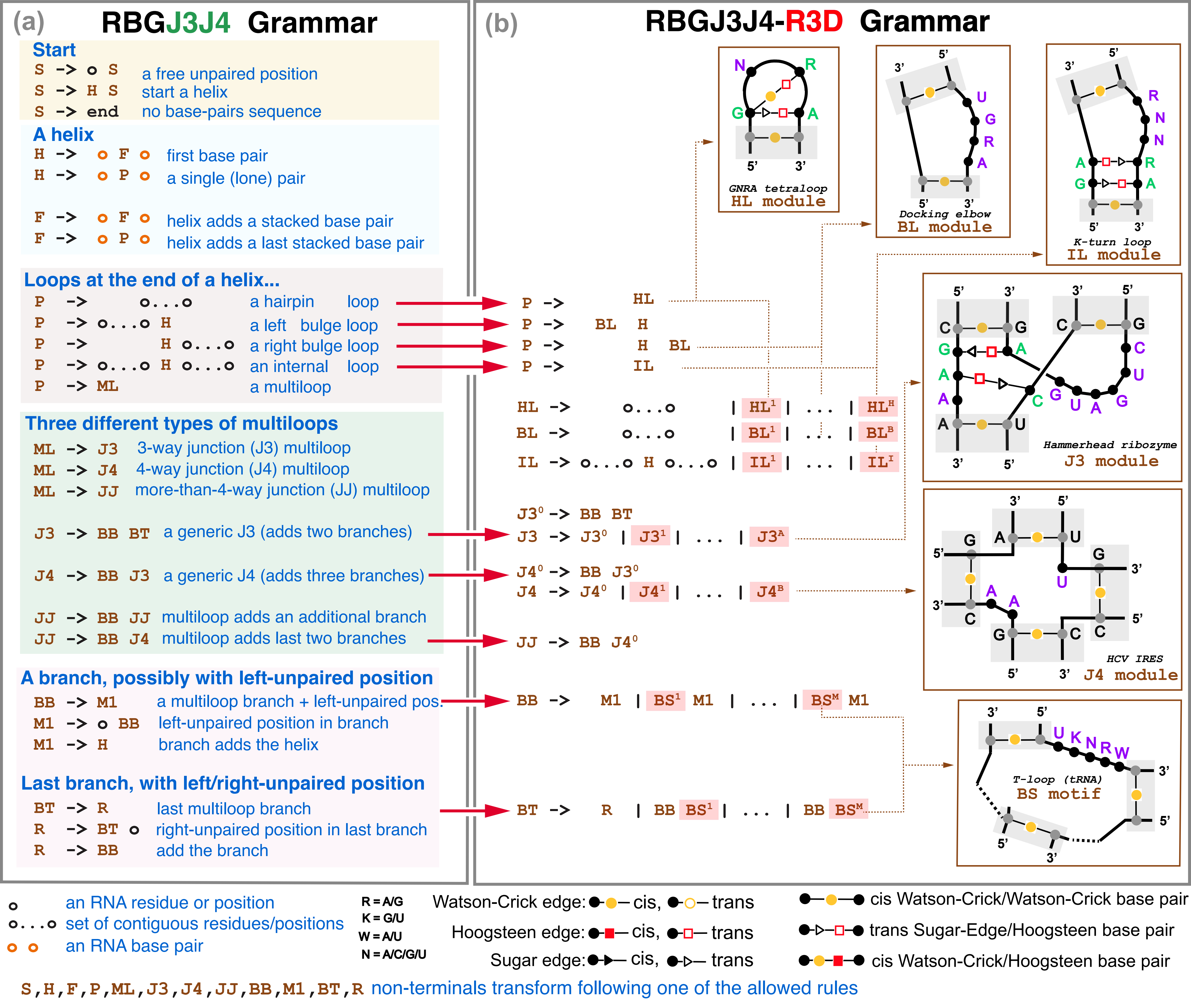

(a) RBGJ3J4 Grammar
Start
S -> o S a free unpaired position
S -> H S start a helix
S -> end no base-pairs sequence
A helix
H -> o F o first base pair
H -> o P o a single (lone) pair
F -> o F o helix adds a stacked base pair
F -> o P o helix adds a last stacked base pair
Loops at the end of a helix...
P -> o...o a hairpin loop
P -> o...o H a left bulge loop
P -> H o...o a right bulge loop
P -> o...o H o...o an internal loop
P -> ML a multiloop
Three different types of multiloops
ML -> J3 3-way junction (J3) multiloop
ML -> J4 4-way junction (J4) multiloop
ML -> JJ more-than-4-way junction (JJ) multiloop
J3 -> BB BT a generic J3 (adds two branches)
J4 -> BB J3 a generic J4 (adds three branches)
JJ -> BB JJ multiloop adds an additional branch
JJ -> BB J4 multiloop adds last two branches
A branch, possibly with left-unpaired position
BB -> M1 a multiloop branch + left-unpaired pos.
M1 -> o BB left-unpaired position in branch
M1 -> H branch adds the helix
Last branch, with left/right-unpaired position
BT -> R last multiloop branch
R -> BT o right-unpaired position in last branch
R -> BB add the branch
(b) RBGJ3J4-R3D Grammar
P -> HL
P -> BL H
P -> H BL
P -> IL
HL -> o...o | HL^1 | ... | HL^H
BL -> o...o | BL^1 | ... | BL^B
IL -> o...o H o...o | IL^1 | ... | IL^I
$J3^0$ -> BB BT
J3 -> $J3^0$ | $J3^1$ | ... | $J3^A$
$J4^0$ -> BB $J3^0$
J4 -> $J4^0$ | $J4^1$ | ... | $J4^B$
JJ -> BB $J4^0$
BB -> M1 | BS^1 M1 | ... | BS^M M1
BT -> R | BB BS^1 | ... | BB BS^M
GNRA tetraloop
HL module
Docking elbow
BL module
K-turn loop
IL module
Hammerhead ribozyme
J3 module
HCV IRES
J4 module
T-loop (tRNA)
BS motif
o an RNA residue or position
o...o set of contiguous residues/positions
o o an RNA base pair
R = A/G
K = G/U
W = A/U
N = A/C/G/U
Watson-Crick edge: cis, trans
Hoogsteen edge: cis, trans
Sugar edge: cis, trans
cis Watson-Crick/Watson-Crick base pair
trans Sugar-Edge/Hoogsteen base pair
cis Watson-Crick/Hoogsteen base pair
S,H,F,P,ML,J3,J4,JJ,BB,M1,BT,R non-terminals transform following one of the allowed rules
